# Outcomes of surgery for unstable odontoid fractures combined with instability of adjacent segments

**DOI:** 10.1186/s13018-014-0064-9

**Published:** 2014-08-28

**Authors:** Lei Wang, Chao Liu, Qing-hua Zhao, Ji-Wei Tian

**Affiliations:** 1 Department of Orthopedics, Shanghai Jiaotong University Affiliated the first people’s Hospital, No.1878 North Sichuan Road, Shanghai 200080, China

**Keywords:** Odontoid fractures, Adjacent section, Instability, Operative strategies, Surgical treatment

## Abstract

**Background:**

At present, traumatic atlantoaxial dislocation or C2-3 instability complicating odontoid fractures remains rarely reported. The aim of this study was to further investigate the surgical treatment strategies and curative effects for odontoid fractures combined with instability of adjacent segments.

**Methods:**

This is a retrospective study of 12 patients (5 females and 7 males; age, 21–65 years) who underwent internal fixation for odontoid fractures (type II and shallow type III) and atlantoaxial instability in 6 cases, C2-3 instability in 4 cases, simultaneous C1-2 and C2-3 instability in 2 cases between January 2005 and June 2012. Accordingly, individualized surgeries were performed. Fracture healing and bone fusion were determined on X-ray scan. Upper limbs, lower limbs and sphincter functions were assessed using the Japanese Orthopaedic Association (JOA) score. Frankel grading system was used for the evaluation of neurological situation.

**Results:**

Mean follow-up time of all 12 cases was 16.4 months (range, 12 to 48 months). Odontoid fracture healing was obtained in all patients within 9 months, and graft fusion was achieved within 6 months. JOA score was significantly improved from 6.3 ± 3.1 preoperatively to 11.1 ± 4.6 at 12 months after operation (*P* = 0.007), with 50.5 ± 25.7% recovery rate and 66.7% excellent and good rate. Except one patient still had Frankel grade B neurological injury at 12 months after surgery, the other patients improved their neurological situation (at 1 grade in Frankel scale). One patient developed wound fat liquefaction which resolved by changing the dressing. Cerebrospinal fluid leakage occurred in three patients, which resolved after the continuous drainage for 2 days.

**Conclusions:**

According to the characteristics of odontoid fractures, the individualized operative procedure should be performed, resulting in high fracture healing rate, function recovery rate, and less, transient complications.

## Background

Anatomically, the odontoid process, with its attached ligamentous ring formed ventrally by the anterior arch of the atlas and posteriorly by the transverse atlantal ligament, is the keystone to maintain the stability of atlantoaxial articulation [1],[2]. Any disruption of the odontoid process or the ligaments will predispose the patients to atlantoaxial joint instability (e.g., dislocation or subluxation) which further causes compression of the spinal cord, nerve root, or vertebral artery [3], ultimately leading to neurological damages or death [4]. In addition, recent studies also reported the cases who sustained odontoid fractures combined with instability of C2-3 [5]-[7]. The multiple fractures make their management challenging for surgeons. At present, traumatic atlantoaxial dislocation [8]-[10] or spondylolisthesis of C2–3 [5]-[7] complicating odontoid fractures remains rarely reported. This study was a retrospective review of 12 patients who suffered odontoid fractures combined with atlantoaxial instability, C2–3 instability, or C1–2 and C2–3 simultaneous instability. We aimed to discuss the management of these types of injury based in our experience.

## Materials and methods

### Patients

From January 2005 to June 2012, 12 patients (7 males and 5 females; average age, 36 years, range, 21–65 years) suffered odontoid fractures combined with instability of adjacent segments and underwent internal fixation in our hospital. The combined instability of adjacent segments included atlantoaxial instability in 6 cases (atlantoaxial dislocation/subluxation in 5 cases and atlas fracture in 1 case), C2–3 instability in 4 cases (C2-3 disc injury in 2 cases, Hangman fracture in 1 case and C3 fracture in 1 case), and C1–2 and C2–3 instability in 2 cases (atlantoaxial dislocation with C2–3 disc injury in 1 case and atlas fracture with C2/3 dislocation in 1 case) (see Table 1). The injury mechanisms were traffic accidents in 8 cases and falling from a height in 4 cases. The upper cervical spine deformities and degenerative diseases were not involved in them. The mainly clinical symptoms were occipital pain, limited neck mobility, torticollis with limited neck movements, numbness in the upper extremity, inability to exercise for lower extremity, and other neurological symptoms. Odontoid fractures were diagnosed by anteroposterior, lateral, and open mouth (atlantoaxial) X-rays, computed tomography (CT) scanning, three-dimensional (3-D CT) scanning, and magnetic resonance imaging (MRI). There were 9 type II and 3 shallow type III fractures according to the classification of Anderson and D’Alonzo [11]. The type II and rostral ‘shallow’ type III odontoid fractures represent highly unstable entities [12]. Twelve patients manifested nerve injury (Frankel grade A in 1 case, grade B in 2 cases, grade C in 3 cases, and grade D in 6 cases) [13]. Due to the retrospective nature of the study, no further approvals of the patient or the local ethics committee were necessary.

**Table 1:** Thirty-seven odontoid fractures undergoing surgical fixation

**Cases**	**Sex**	**Age**	**Type of odontoid fractures**	**Combined injuries**	**Injury mechanism**	**Pre-nerve injury**	**Time to surgery (d)**	**Surgery method**	**Time of surgery (min)**	**Graft fusion time (mon)**	**Fracture healing time (mon)**	**Complication**	**Post-nerve injury**	**Pre-JOA**	**Post-JOA**	**Recovery rate (%)**
1	M	45	II	Atlantoaxial dislocation	Traffic accidents	C	1	Anterior screw fixation + posterior atlantoaxial screw fixation	120	-	3	No	D	6	15	81.8
2	M	48	II	Atlantoaxial dislocation	Traffic accidents	D	2	Anterior screw fixation + posterior atlantoaxial screw fixation	150	-	3	No	E	8	14	66.7
3	M	52	Shallow III	Atlantoaxial subluxation	Falling from a height	C	4	Anterior screw fixation + posterior atlantoaxial screw fixation	160	-	4	No	D	5	9	33.3
4	F	65	II	Atlantoaxial subluxation	Traffic accidents	D	3	Posterior atlantoaxial screw fixation	110	-	6	No	E	8	15	77.8
5	F	60	II	Atlantoaxial subluxation	Traffic accidents	C	3	Posterior atlantoaxial screw fixation	100	-	6	No	E	7	12	50
6	M	27	II	Atlas fracture	Falling from a height	D	2	Occipital cervical fusion	120	-	4.5	Leakage of cerebrospinal fluid	E	10	15	71.4
7	M	38	Shallow III	Hangman fracture	Traffic accident	B	3	Anterior cervical CAGE and plate fixation	80	3	3	No	B	3	4	7.1
8	F	42	Shallow III	C3 fracture	Traffic accidents	D	3	Posterior C2, C3 fixation	90	6	9	Fat liquefaction	E	8	13	55.6
9	M	53	II	C2–3 disc injury	Falling from a height	D	4	Anterior cervical CAGE and plate fixation	100	4.5	4.5	No	E	9	13	50
10	M	60	II	C2–3 disc injury	Traffic accidents	D	3	Anterior cervical CAGE and plate fixation	90	6	6	No	E	9	15	75
11	F	32	II	Atlantoaxial dislocation + C2–3 disc injury	Falling from a height	B	3	C1–C3 fixation	130	6	6	Leakage of cerebrospinal fluid	C	2	5	20
12	F	21	II	Atlas fracture + C2/3 dislocation	Traffic accidents	A	7	Anterior and posterior surgery	150	6	6	Leakage of cerebrospinal fluid	B	0	3	17.6

*JOA* Japanese Orthopaedic Association.

### Surgical strategies

All the 12 patients underwent internal fixation surgery at 1–7 days after injury, with an average of 3.2 days. According to the characteristics of odontoid fractures and the stable condition of atlantoaxial joint and C2–3, we applied different surgical methods as follows (see Table 1). The patients with simple odontoid fractures were treated by anterior odontoid screw fixation. If it was difficult for odontoid screw placement, posterior fixation was recommended. For odontoid fractures with atlantoaxial dislocation or atlas fractures, posterior atlantoaxial pedicle screw fixation or occipital cervical fusion was carried out if clinically indicated. For odontoid fractures with C2–3 disc injury/Hangman fracture, the anterior C2/3 discectomy, interbody fusion, and anterior cervical plate fixation were used. For odontoid fractures with C3 fractures, posterior C2–3 pedicle screw or lateral mass screw fixation were elected. For odontoid fractures with C1/2 and C2/3 instability, posterior C1–3 pedicle screw/lateral mass screw fixation or combined with anterior surgery was employed. If atlantoaxial pedicle screw placement was difficult, occipitocervical fusion was used. Besides, no significant transverse ligament rupture was observed via MRI in six cases with odontoid fractures and atlantoaxial dislocation/fracture atlas. Thus, interbody fusion was not performed for them. The remaining patients underwent anterior and/or posterior interbody fusion.

After operation, a drainage tube was placed for 24 to 48 h and antibiotics were routinely used for 1 to 3 days. At 12–14 days after operation, the sutures were removed and a plastic cervical gear was used for protection for 3 months. Follow-up visits were scheduled at 6 weeks, 3, 6, and 12 months after surgery.

### Outcome measures

Fracture healing was defined as trabecular bridging the fracture and faint fracture line on the cervical spine X-ray [14],[15]. Bone fusion was judged by less than 2° of movement between the spinous processes on flexion-extension lateral radiographs [16]. Movement of ≥2° on flexion/extension radiographs was considered and regarded as a pseudarthrosis [17]. If pseudarthrosis could not be identified or excluded, CT scan was performed to evaluate about fusion. The Japanese Orthopaedic Association (JOA) scoring system for cervical myelopathy [18] (Table 2) was used to evaluate the treatment effects at 12 months after surgery compared with before operation. Recovery rate was calculated by the following formula: (postoperative score − preoperative score) / (17 [full score] − preoperative score) × 100%. A recovery rate greater than 75% was graded as excellent, 50% to 75% as good, 25% to 50% as fair, and less than 25% as poor [19].

**Table 2:** Japanese Orthopaedics Association score for cervical myelopathy

**Function**	**Score**	**Remarks**
Motor function of upper extremity	0	Unable to eat with either spoon or chopsticks
	1	Possible to eat with spoon, but not chopsticks
	2	Possible to eat with chopsticks, but inadequate
	3	Possible to eat with chopsticks, but awkward
	4	Normal
Motor function of lower extremity	0	Impossible to walk
	1	Need cane or aid on flat ground
	2	Need cane or aid only on stairs
	3	Possible to walk without cane or aid, but slow
	4	Normal
Sensory		
Upper extremity	0	Apparent sensory loss
	1	Minimal sensory loss
	2	Normal
Lower extremity	0	Apparent sensory loss
	1	Minimal sensory loss
	2	Normal
Trunk	0	Apparent sensory loss
	1	Minimal sensory loss
	2	Normal
Sphincter dysfunction	0:	Complete urinary retention
	1	Severe disturbance
	2	Mild disturbance
	3	Normal

### Statistical methods

All data were analyzed by Microsoft Excel 2010 (Microsoft, Redmond, Washington) and using SPSS10.0 software (SPSS Inc., Chicago, IL, USA). The difference between preoperative and postoperative JOA score was analyzed by paired *t* test. *P* < 0.05 was considered statistically significant.

## Results

**Figure 1:**
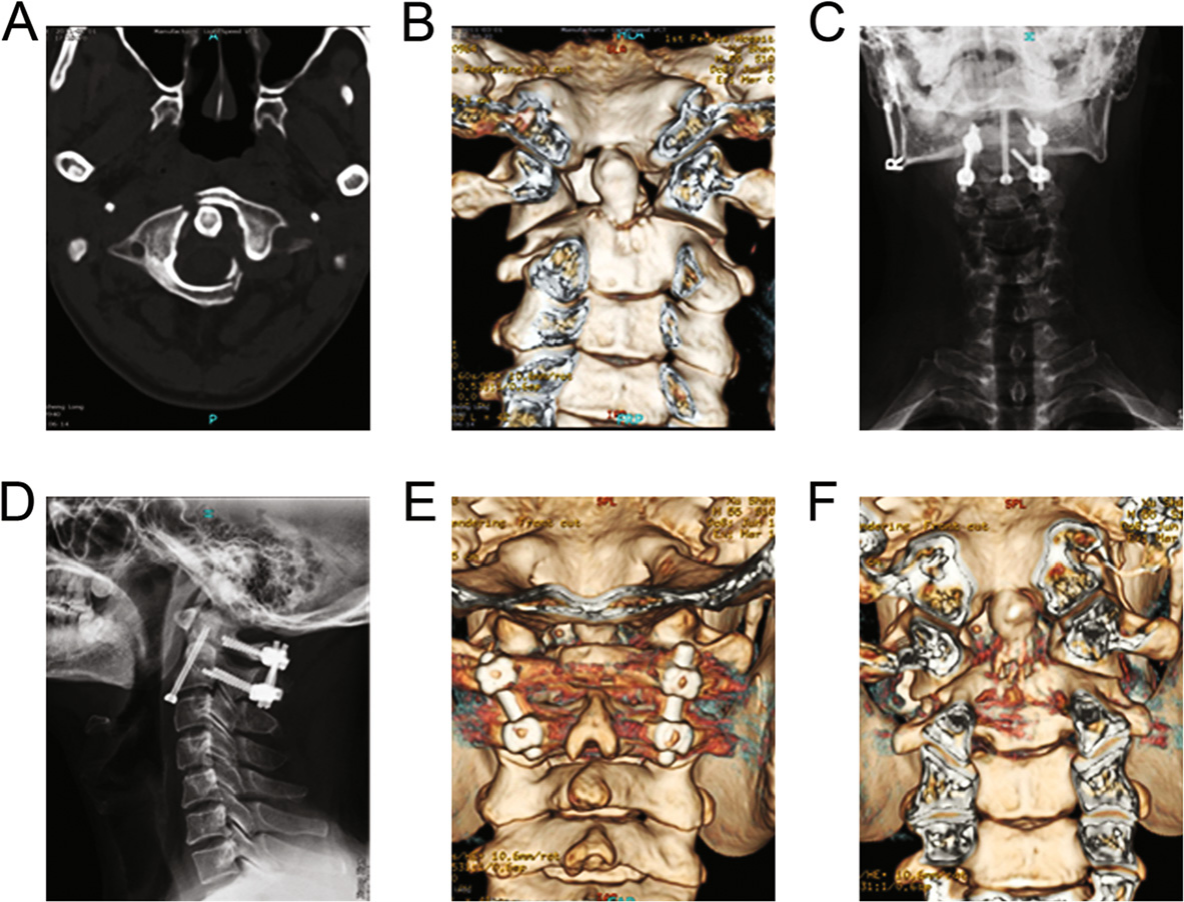
**A 34-year-old male patient developed pain symptoms, limited neck movement, and numbness of his two upper extremities after traffic accidents.** CT scanning **(A)** and 3-D CT **(B)** indicated fractures of the odontoid process of the axis and C1 before operation. Anteroposterior **(C)** and lateral **(D)** cervical spine X-ray scanning showed a fracture line in the odontoid anterior after anterior screw and posterior atlantoaxial pedicle screw fixation. 3-D CT showed bone fusion **(E)** and fracture healing **(F)** at 6 months after operation.

**Figure 2:**
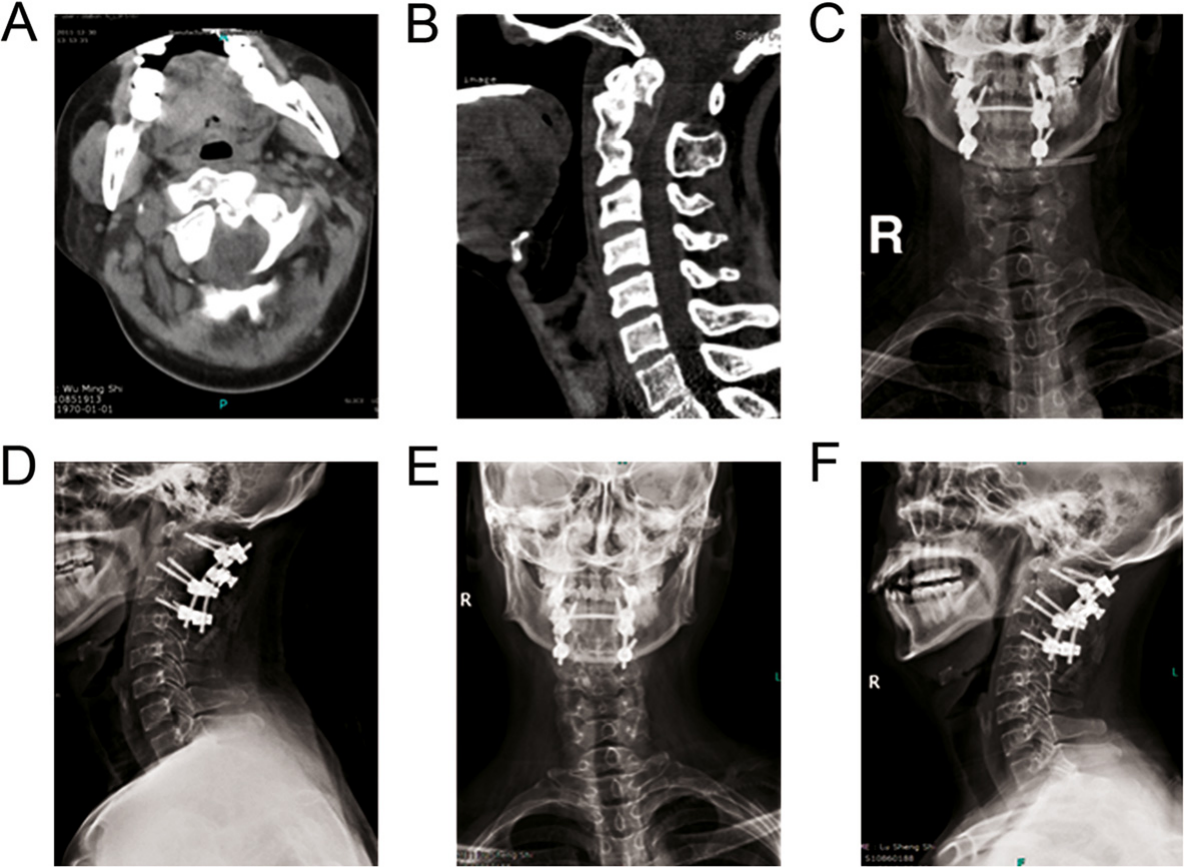
**A 43-year-old male patient developed pain symptoms and paralysis of two lower extremities by traffic accidents.** CT scanning indicated fractures of the odontoid process of the axis **(A)**, combined with C1–2 and C2–3 dislocation **(B)**. Anteroposterior **(C)** and lateral **(D)** cervical spine X-ray scanning showed excellent reduction after posterior C1–2 cervical pedicle screw and C3 lateral mass screws fixation. At 1 year after operation, the anteroposterior **(E)** and lateral **(F)** cervical spine X-ray scanning suggested bone fusion and no displacement of the implant.

Mean follow-up time of all 12 cases was 16.4 months (12 to 48 months). All the preoperative symptoms, including occipital pain, limited neck activity, and torticollis were favorably resolved after operation. On X-ray scan, odontoid fracture union was obtained in all patients within 9 months and bone fusion was achieved within 6 months, with an average of 5.3 months. For the 6 cases without interbody fusion, the implants were removed after an average of 9 months in order to restore atlantoaxial rotation function. The straight alignment of the cervical spine was maintained throughout the follow-up period. No internal fixation loosening, extrusion or breakage, secondary vertebral artery, or nerve damage was observed. One patient developed wound fat liquefaction which was resolved by changing the dressing. Cerebrospinal fluid (CSF) leakage occurred in three patients, which was healed after the continuous drainage for 2 days. JOA score was significantly improved from 6.3 ± 3.1 preoperatively to 11.1 ± 4.6 at 12 months after operation (*P* = 0.007), with 50.5 ± 25.7% recovery rate and 66.7% excellent and good rate. Except 1 patient still had Frankel grade B neurological injury at 12 months after surgery; the other patients improved their neurological situation (see Table 1). The typical cases are shown in Figures 1 and 2.

## Discussion

Odontoid fracture is a common traumatic upper cervical spine injury, accounting for 10%–14% of all cervical spine fractures [20]. However, the combination of atlantoaxial instability, C2–3 instability, or simultaneous C1–2 and C2–3 instability with odontoid fractures is rare. In this study, we reported the individualized operative procedures for those patients and obtained the excellent outcome.

Anterior odontoid screw fixation is an effective surgical approach for type II and type III odontoid fractures. This method can provide immediate stabilization, cause less postoperative pain, require no bone graft and preserves normal atlantoaxial rotational motion [21],[22]. Therefore, anterior odontoid screw fixation was preferentially considered for our patients. However, if only the odontoid fractures were reduced, the remaining atlantoaxial, C2–3, or simultaneous C1–2 and C2–3 instability could lead to high cervical spinal cord injury, resulting in quadriplegia or even death. Hence, we suggest to simultaneously restoring C1/2/3 stability and odontoid fractures.

Due to the ease of dissection, the posterior approach has been commonly used to stabilize the C1–C2. Previous studies have demonstrated that pedicle screws can maintain a higher rotational and lateral bending stiffness and withstand higher toggle forces (or pullout strength) compared to the intralaminar screws [23], pars screws [24],[25], and lateral mass screws [26]. Thus, pedicle screws may be the most biomechanically stable for atlantoaxial fixation. In this study, we also attempted to reduce the atlantoaxial dislocation or subluxation through posterior pedicle screws fixation and achieved the favorable outcomes, with the highest recovery rate up to 81.8% and without complication (e.g., internal fixation loosening, extrusion, or breakage).

If it is impossible to place screws into C1–C2 due to atlas fracture, an occipitocervical fixation is usually advocated [9],[27]. However, this approach does not provide good exposure of the articular processes due to the anatomical characteristics of occipitocervical region, and the dura is easily to be injured during surgery, leading to the development of CSF leakage [9],[28]. In this study, one patient underwent occipitocervical fusion and presented postoperative CSF leakage. However, this complication was transient and resolved after the continuous drainage for 2 days.

It is technically feasible to treat C2–C3 instability by anterior C2–3 discectomy followed by an interbody fusion and anterior cervical plate fixation or posterior C2–3 pedicle screw or lateral mass screw fixation [6],[29],[30]. However, anterior stabilization in these injuries may be mandatory from a biomechanical analysis [31]. The anterior approach not only clears the damaged disc directly to relieve spinal cord compression, restore spinal sequence, and reconstruct C2/3 stability but also causes less injuries to spinal cord and artery [32]. If spinal cord compression is from the posterior, a posterior approach should be performed. In the present study, three patients underwent anterior cervical plate fixation and one underwent posterior C2–3 pedicle screw and lateral mass screw fixation. Although the fractures were all healed after 6 months, the JOA (7.1% recovery rate) and Frankel grade (still B) were not significantly improved in the patient with Hangman’s fracture. We believe that this may be attributed to the serious nerve injury preoperatively and thus suggest the surgery should be performed as early as possible. In addition, fat liquefaction was observed in one patient, which delayed the fracture healing and bone fusion time.

We also reported two patients who sustained simultaneous C1–2 and C2–3 instability with odontoid fractures, indicating that the C1–3 should be fixed. Previous study recommended to use the C1–3 lateral mass screw fixation [33] which provides a better immobilization, anti-fatigue, and anti-subsidence effects. However, this method is difficult to operate and requires imaging equipment to monitor, likely resulting in vertebral artery injury. However, no bleeding or infection was observed in our two patients, but postoperative CSF leakage occurred. We speculate that this may result from the mesh-like holes in the dura because of the long-term severe compression [28].

However, the study has some potential limitations. Firstly, as a retrospective study, patients were not randomly assigned to a surgical procedure. The choice of surgery might be biased by the surgeons’ preference based on the preoperative condition of the patient. Secondly, because the combination of atlantoaxial instability, C2–3 instability, or simultaneous C1–2 and C2–3 instability with odontoid fractures is rare, it was difficult to obtain a sufficient number of patients. Thirdly, the follow-up period was short. Therefore, future studies with large sample size and longer-term monitoring need to be performed to verify our results.

## Conclusions

Overall, our study reported cases who suffered odontoid fractures combined with atlantoaxial instability, C2–3 instability, or simultaneous C1–2 and C2–3 instability and investigated the individualized operative procedures for them. Although there were transient complications (e.g., CSF leakage or fat liquefaction) postoperatively, all the fractures were favorably healed, the JOA and neurological situation were significantly improved. These indicate that our surgical strategies may be reasonable, but further randomized controlled studies with large sample size are still needed to confirm our conclusion.

## Competing interests

The authors declare that they have no competing interests.

## Authors’ contributions

LW and CL carried out the design and coordinated the study, participated in most of the experiments, and prepared the manuscript. QZ provide assistance in the design of the study, coordinated and carried out all the experiments, and participated in manuscript preparation. JWT provided assistance for all experiments. All authors have read and approved the content of the manuscript.

## References

[B1] Meng H Gao Y Li M Luo Z Du J Posterior atlantoaxial dislocation complicating odontoid fracture without neurologic deficit: a case report and review of the literature Skeletal Radiol 2014 43 7 1001 1006 10.1007/s00256-013-1809-y 24469150

[B2] Scott EW Haid RW Jr Peace D Type I fractures of the odontoid process: implications for atlanto-occipital instability: case report J Neurosurg 1990 72 3 488 492 10.3171/jns.1990.72.3.0488 2303882

[B3] Steel H Anatomical and mechanical considerations of atlanto-axial articulations Journal of Bone and Joint Surgery-American volume 1968 Journal Bone Joint Surgery Inc., Needham, MA 02192 1481-&

[B4] Evaniew N, Yarascavitch B, Madden K, Ghert M, Drew B, Bhandari M, Kwok D: **Atlantoaxial instability in acute odontoid fractures is associated with nonunion and mortality.** *Spine J* 2014. doi:10.1016/j.spinee.2014.03.029. 10.1016/j.spinee.2014.03.029 24662216

[B5] Blondel B Metellus P Fuentes S Dutertre G Dufour H Single anterior procedure for stabilization of a three-part fracture of the axis (odontoid dens and hangman fracture): case report Spine 2009 34 7 E255 E257 10.1097/BRS.0b013e318195ab2d 19333089

[B6] Koller H Assuncao A Kammermeier V Holz U Simultaneous anterior arthrodesis C2-3 and anterior odontoid screw fixation for stabilization of a 4-part fracture of the axis—a technical description J Spinal Disord Tech 2006 19 5 362 367 10.1097/01.bsd.0000204502.99471.9a 16826010

[B7] Shinbo J Sameda H Ikenoue S Takase K Yamaguchi T Hashimoto E Enomoto T Kanazuka A Mimura M Simultaneous anterior and posterior screw fixations confined to the axis for stabilization of a 3-part fracture of the axis (odontoid, dens, and hangman fractures: report of 2 cases J Neurosurg Spine 2014 20 3 265 269 10.3171/2013.12.SPINE12448 24409982

[B8] Riouallon G Pascal-Moussellard H Atlanto-axial dislocation complicating a type II odontoid fracture. Reduction and final fixation Orthop Traumatol Surg Res 2014 100 3 341 345 10.1016/j.otsr.2013.12.026 24725907

[B9] Moreau P Nguyen V Atallah A Kassab G Thiong’o M Laporte C Traumatic atlantoaxial dislocation with odontoid fracture: a case report Orthop Traumatol Surg Res 2012 98 5 613 617 10.1016/j.otsr.2012.03.012 22901523

[B10] Spoor AB Diekerhof CH Bonnet M Öner FC Traumatic complex dislocation of the atlanto-axial joint with odontoid and C2 superior articular facet fracture Spine 2008 33 19 E708 E711 10.1097/BRS.0b013e31817c140d 18758352

[B11] Anderson LD D’ALONZO RT Fractures of the odontoid process of the axis J Bone Joint Surg 1974 56 8 1663 1674 4434035

[B12] Wang J Zhou Y Zhang ZF Li CQ Zheng WJ Liu J Comparison of percutaneous and open anterior screw fixation in the treatment of type II and rostral type III odontoid fractures Spine 2011 36 18 1459 1463 10.1097/BRS.0b013e3181f46ee8 21240052

[B13] Frankel H Hancock D Hyslop G Melzak J Michaelis L Ungar G Vernon J Walsh J The value of postural reduction in the initial management of closed injuries of the spine with paraplegia and tetraplegia Spinal Cord 1969 7 3 179 192 10.1038/sc.1969.30 5360915

[B14] Dijkman BG Sprague S Schemitsch EH Bhandari M When is a fracture healed? Radiographic and clinical criteria revisited J Orthop Trauma 2010 24 S76 S80 10.1097/BOT.0b013e3181ca3f97 20182242

[B15] Bhandari M Chiavaras M Ayeni O Chakraverrty R Parasu N Choudur H Bains S Sprague S Petrisor B Assessment of radiographic fracture healing in patients with operatively treated femoral neck fractures J Orthop Trauma 2013 27 9 e213 e219 10.1097/BOT.0b013e318282e692 23287749

[B16] Hacker RJ Cauthen JC Gilbert TJ Griffith SL A prospective randomized multicenter clinical evaluation of an anterior cervical fusion cage Spine 2000 25 20 2646 2655 10.1097/00007632-200010150-00017 11034651

[B17] Yu S Li F Yan N Yuan C He S Hou T Anterior fusion technique for multilevel cervical spondylotic myelopathy: a retrospective analysis of surgical outcome of patients with different number of levels fused PloS one 2014 9 3 e91329 10.1371/journal.pone.0091329 24618678 PMC3949986

[B18] Takagishi N Nobuhara K Fukuda H Matsuzaki A Mikasa M Yamamoto R Shoulder evaluation sheet J Jpn Orthop Assoc 1987 61 623

[B19] Kawaguchi Y Matsui H Ishihara H Gejo R Yasuda T Surgical outcome of cervical expansive laminoplasty in patients with diabetes mellitus Spine 2000 25 5 551 555 10.1097/00007632-200003010-00004 10749630

[B20] Debernardi A D’Aliberti G Talamonti G Villa F Piparo M Cenzato M Traumatic (type II) odontoid fracture with transverse atlantal ligament injury: a controversial event World Neurosurg 2013 79 779 783 10.1016/j.wneu.2012.01.055 22381856

[B21] Song K-J Lee K-B Kim K-N Treatment of odontoid fractures with single anterior screw fixation J Clin Neurosci 2007 14 9 824 830 10.1016/j.jocn.2006.06.016 17660055

[B22] Lee S-C Chen J-F Lee S-T Management of acute odontoid fractures with single anterior screw fixation J Clin Neurosci 2004 11 8 890 895 10.1016/j.jocn.2004.03.023 15519869

[B23] Lapsiwala SB Anderson PA Oza A Resnick DK Biomechanical comparison of four C1 to C2 rigid fixative techniques: anterior transarticular, posterior transarticular, C1 to C2 pedicle, and C1 to C2 intralaminar screws Neurosurgery 2006 58 3 516 521 10.1227/01.NEU.0000197222.05299.31 16528192

[B24] Su BW Shimer AL Chinthakunta S Salloum K Ames CP Vaccaro AR Bucklen B Comparison of fatigue strength of C2 pedicle screws, C2 pars screws, and a hybrid construct in C1–C2 fixation Spine 2014 39 1 E12 E19 10.1097/BRS.0000000000000063 24108297

[B25] Dmitriev AE Lehman RA Jr Helgeson MD Sasso RC Kuhns C Riew DK Acute and long-term stability of atlantoaxial fixation methods: a biomechanical comparison of pars, pedicle, and intralaminar fixation in an intact and odontoid fracture model Spine 2009 34 4 365 370 10.1097/BRS.0b013e3181976aa9 19214095

[B26] Fensky F Kueny RA Sellenschloh K Püschel K Morlock MM Rueger JM Lehmann W Huber G Hansen-Algenstaedt N Biomechanical advantage of C1 pedicle screws over C1 lateral mass screws: a cadaveric study Eur Spine J 2014 23 4 724 731 10.1007/s00586-013-3143-4 24378628 PMC3960438

[B27] Dai L Yuan W Ni B Liu H Jia L Zhao D Xu Y Surgical treatment of nonunited fractures of the odontoid process, with special reference to occipitocervical fusion for unreducible atlantoaxial subluxation or instability Eur Spine J 2000 9 2 118 122 10.1007/s005860050221 10823427 PMC3611364

[B28] He B Yan L Xu Z Chang Z Hao D The causes and treatment strategies for the postoperative complications of occipitocervical fusion: a 316 cases retrospective analysis Eur Spine J 2014 28 8 1 5 10.1007/s00586-014-3354-3 24838504

[B29] Song J Taghavi CE Hsu DW Song K-J Song J-H Lee K-B Radiological changes in anterior cervical discectomy and fusion with cage and plate construct: the significance of the anterior spur formation sign Spine 2012 37 4 272 279 10.1097/BRS.0b013e31821c3cbf 21508883

[B30] Xie N Khoo LT Yuan W Ye X-J Chen D-Y Xiao J-R Ni B Combined anterior C2-C3 fusion and C2 pedicle screw fixation for the treatment of unstable Hangman’s fracture: a contrast to anterior approach only Spine 2010 35 6 613 619 10.1097/BRS.0b013e3181ba3368 20150833

[B31] Arand M Neller S Kinzl L Claes L Joachim Wilke H The traumatic spondylolisthesis of the axis: a biomechanical in vitro evaluation of an instability model and clinical relevant constructs for stabilization Clin Biomech 2002 17 6 432 438 10.1016/S0268-0033(02)00037-2 12135544

[B32] Tuite GF Papadopoulos SM Sonntag VK Caspar plate fixation for the treatment of complex hangman’s fractures Neurosurgery 1992 30 5 761 764 10.1227/00006123-199205000-00019 1584391

[B33] Horn EM Hott JS Porter RW Theodore N Papadopoulos SM Sonntag VK Atlantoaxial stabilization with the use of C1-3 lateral mass screw fixation: technical note J Neurosurg Spine 2006 5 2 172 177 10.3171/spi.2006.5.2.172 16925087

